# Enhanced Antibacterial Activity of Novel Fluorescent Glutathione-Capped Ag Nanoclusters

**DOI:** 10.3390/ijms24098306

**Published:** 2023-05-05

**Authors:** Roman Tumskiy, Boris Khlebtsov, Anastasiia Tumskaia, Stella Evstigneeva, Evgeniya Antoshkina, Andrey Zakharevich, Nikolai G. Khlebtsov

**Affiliations:** 1 Institute of Biochemistry and Physiology of Plants and Microorganisms, Saratov Scientific Centre of the Russian Academy of Sciences (IBPPM RAS), 410049 Saratov, Russia; tumskiyr@gmail.com (R.T.);; 2 Institute of Physics, Saratov State University, 410012 Saratov, Russia; 3 A.N. Nesmeyanov Institute of Organoelement Compounds of Russian Academy of Sciences (INEOS RAS), 28 Vavilova Str, Bld.1, 119334 Moscow, Russia; 4 Moscow Institute of Physics and Technology, National Research University, 9 Institutskiy per., 141700 Dolgoprudny, Russia

**Keywords:** Ag nanoclusters, antibacterial activity, fluorescence, molecular docking

## Abstract

Ag nanomaterials are promising candidates for the discovery of next-generation antibiotics with a high antibacterial effect against multi-drug resistant strains. This paper reports a simple synthesis of novel water-soluble glutathione-capped silver nanoclusters (GSH-Ag NCs) with an enhanced antibacterial activity. According to thin layer chromatography (TLC), the synthesized GSH-Ag NCs are an individual fraction of the same composition without any impurities. According to matrix-assisted laser desorption ionization mass spectrometry (MALDI-MS) and energy dispersive X-ray (EDX) analyses, the silver core of the GSH-Ag NCs contains approximately 35 silver atoms, and the molecular weight of these nanoclusters is about 11 kDa. The fabricated silver nanoclusters have a reddish fluorescence (λex/λem = 509/645 nm), with a large Stokes shift (>130 nm), and ultra-small size (less than 2 nm) according to transmission electron microscopy (TEM) data and dynamic light scattering (DLS) analysis. The antibacterial activity and minimal inhibitory concentrations of the silver nanoclusters towards *Escherichia coli*, *Staphylococcus aureus*, *Bacillus cereus* and *Enterobacter cloacae* were evaluated using the agar well-diffusion method and resazurin metabolism assay. The antibacterial activity of chelated silver in the nanoclusters was found to be significantly higher compared to the activity of free silver ions. To explain the possible mechanisms underlying the antibacterial actions of the GSH-Ag nanoclusters, molecular docking was performed, and prospective bacterial targets were identified using AutoDock.

## 1. Introduction

Since ancient times, silver has been known to be effective against a wide spectrum of bacteria [1]. Today, silver-based materials are widely used to inhibit bacterial growth in different medical applications, including dental work, catheters, and the healing of burn wounds [2,3]. Importantly, in low concentrations, silver is not toxic for human cells, and hence it can be considered as promising antimicrobial agent [4]. In recent decades, the scientific community has made great efforts on the investigation of the antibacterial activity of silver, but the mechanism of this activity is not yet fully understood [5]. In fact, the mechanism of the antimicrobial effect of silver (Ag^+^ ions) is closely related to their interaction with the thiol groups of bacterial proteins, although different binding sites still remain a possibility [6]. Moreover, Ag^+^ ions are able to inhibit cell division and bacterial growth. In addition, silver ions cause the release of K^+^ ions from bacteria and interact with nucleic acids [7]. However, the direct application of Ag^+^ ions can induce a tremendous silver-mediated oxidative stress on mammalian cells [8].

The ultra-small silver nanoclusters (Ag NCs) as a new type of nanomaterial have attracted great attention as a promising next-generation silver-based antibacterial agent [9]. Moreover, fluorescent silver nanoclusters are widely applied for biosensing, imaging, and detection of various analytes [10,11]. The core of the NCs contains less than a hundred silver atoms, and the size of the core (<3 nm) is comparable to the Fermi wavelength of electrons. In fact, Ag NCs have demonstrated unique physicochemical characteristics, including a high local concentration of silver ions, appropriately timed silver release kinetics, and tunable cellular uptake behaviors [12,13,14]. However, the molecular mechanisms underlying the antimicrobial effect of Ag NCs is unclear. Some authors have proposed that the effect should depend only on Ag^+^ ions released from the NCs surface [15]. On the other hand, it was found that the silver chelates, rather than free Ag^+^ ions, are the responsible agents for exhibiting the antibacterial properties of the metal [16].

Molecular docking is a powerful tool to study ligand-target intermolecular interactions. Docking has been widely employed as a fast and inexpensive method, which analyzes the conformation and orientation (referred together as the “pose”) of ligands into the binding site of a macromolecular target. Moreover, molecular docking predicts the binding affinity for the ligand-target complex. This method is important for medicinal chemistry and is extensively used in different stages of drug design strategies [17,18].

The present work was aimed on the synthesis, characterization, and evaluation of the in vitro antibacterial activity of novel fluorescent GSH-Ag NCs in comparison with the bioactivity of free Ag^+^ ions. Moreover, we performed a molecular docking to explain the possible mechanism underlying the antimicrobial actions of the GSH-Ag NCs, with identification of prospective bacterial targets.

## 2. Results

### 2.1. Synthesis and Characterization of the GSH-Ag NCs

**Figure 1:**
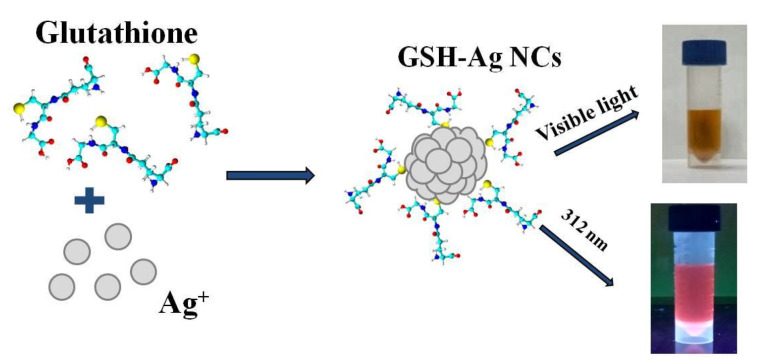
Scheme of the synthesis of the GSH-Ag NCs.

The ultra-small water-soluble glutathione-capped silver nanoclusters (GSH-Ag NCs) were synthesized through a chemical reduction of the intermediate product (silver thiolate) by NaBH4 reagent (Figure 1). In this method, initially silver thiolate is formed due to the reaction between silver nitrate (AgNO_3_) and L-glutathione (GSH). GSH-Ag NCs were formed by the self-assembly of the tripeptide GSH (protecting ligand) monolayer on the silver core, due to the high affinity between the Ag and the SH-groups of GSH [19].

**Figure 2:**
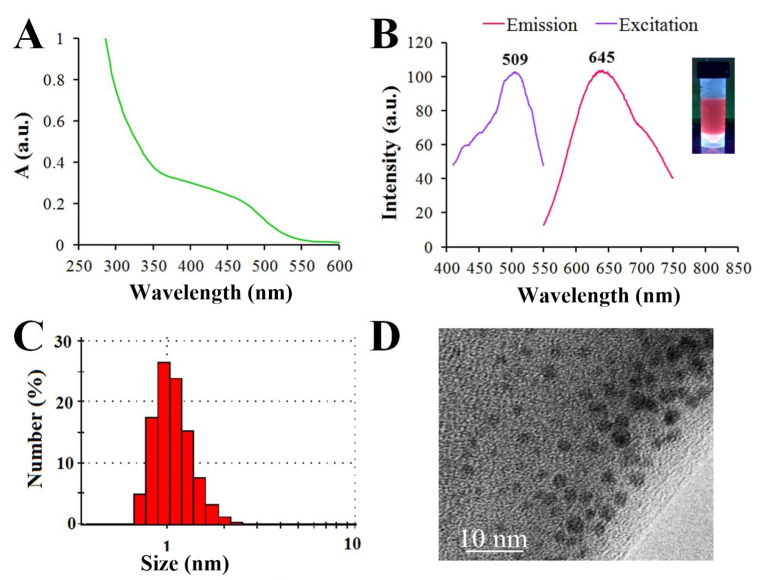
(**A**) UV−visible absorption spectrum of GSH-Ag NCs. (**B**) Photoluminescence spectrum of reddish fluorescent GSH-Ag NCs, showing excitation at 509 nm and emission at 645 nm. (**C**) Dynamic light scattering (DLS) analysis showing a hydrodynamic diameter <2 nm. (**D**) TEM image of the GSH-Ag NCs.

The solution of GSH-Ag NCs in water demonstrated a broad shoulder from 350 to 500 nm in the visible region of absorption spectrum (Figure 2A). As already known, glutathione-capped or other thiol-capped silver nanoclusters exhibit distinct features in the region of 300–800 nm, different from plasmonic nanoparticles [20]. The solutions of GSH-Ag NCs were brownish under visible light and displayed a reddish fluorescence under UV illumination at 312 nm (as shown in Figure 2B). Interestingly, this type of fluorescent silver NCs (λex/λem = 509/645 nm) have a large Stokes shift (>130 nm) (Figure 2B). The quantum yield of GSH-Ag NCs was calculated to be 2.3%, using rhodamine B in ethanol as a reference dye [21]. The hydrodynamic diameter of the GSH-Ag NCs was found to be less than 2 nm according to the DLS data (Figure 2C). The TEM data revealed that the ultra-small size of GSH-Ag NCs, despite being nanoclusters, are sensitive to electron beam exposure, and they gradually convert to larger aggregates or nanoparticles during TEM examination (Figure 2D) [22]. The FTIR spectra of the GSH-Ag NCs revealed that the GSH molecules anchor on the surface of nanoclusters through Ag-S bonding (Figure S1).

**Figure 3:**
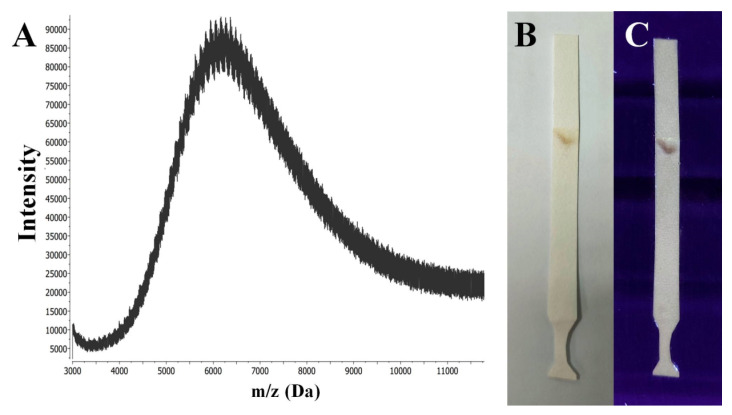
(**A**) MALDI-MS spectra in the positive ion mode of the GSH-Ag NCs sample registered with a linear detector using CHCA (α-cyano-4-hydroxycinnamic acid) as the matrix. (**B**) TLC paper plate after eluting and drying with GSH-Ag NCs (brownish spot) under visible light. (**C**) TLC paper plate with GSH-Ag NCs (brownish violet spot) under UV illumination at 312 nm.

Thin-layer chromatography (TLC) is the simplest of chromatographic techniques for the effective purification and separation of hydrophobic functionalized nanoclusters even with minimal structural differences, such as structural isomers of NCs [23,24]. However, in this work we performed a TLC method for the identification of the fractional composition of the GSH-Ag NCs. To the best of our knowledge, this is the first reported case where a TLC analysis was applied for the characterization of hydrophilic nanoclusters, such as GSH-Ag NCs. From trying various investigated conditions (different solvent systems, sorbents, and variants of TLC analysis), we selected optimal conditions with a high polar solvent system (see experimental part) for the successful eluting and separation of the GSH-Ag NCs. Thus, according to TLC data, the synthesized GSH-Ag NCs are an individual fraction (the retention factor Rf is 0.87), with one composition without any impurities (Figure 3B,C). The next goal for our work was the determination of the GSH-Ag NCs composition (see below).

X-ray crystallography (XRC) is a very robust method for the precise determination of the atomic structure of nanoclusters. However, determining the atomic structure of the NCs is generally very challenging, because the critical step of XRC is growing high-quality single crystals of metal nanoclusters [25]. Unfortunately, in our case, this critical step of crystal growing was not successful for GSH-Ag NCs under various investigated conditions (slow evaporation of NCs solution over the range 4–25 °C in different solvent system: water/ethanol, water/isopropanol, water/acetone, and water/acetonitrile).

Matrix-assisted laser desorption ionization (MALDI) and electrospray ionization (ESI) mass spectrometric analyses have been widely applied to determine the molecular formulas for the family of gold nanoclusters [26]. However, the lower stability of GSH-Ag NCs has proven to be a major limitation in mass spectrometric analyses due to a significant fragmentation of the initial clusters. At the threshold power of the ionizing laser (MALDI-MS), it was not possible to obtain reproducible results, indicating the presence of nanoclusters of a certain composition in the sample. Signals from the sample of the GSH-Ag NCs were obtained using only a high laser power (more than 100 μJ).

The use of such conditions presumably led to a significant fragmentation of the initial clusters, as evidenced by the shape of the signals (as seen in Figure 3A); the collected spectrum shows the presence of a wide series of peaks, with *m*/*z* values ranging from approximately 4000 to 11,000. Furthermore, the distance between adjacent peaks is approximately of *m*/*z* 108 on average, which may indicate the loss of silver atoms by the cluster. Thus, based on routine interpretation of the MALDI-MS data, GSH-Ag NCs should contain approximately 25–35 silver atoms in the core with a molecular weight of about 11 kDa.

**Figure 4:**
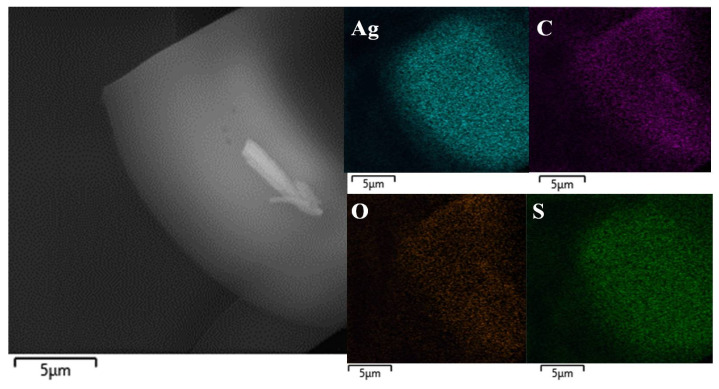
A representative SEM image (**left panel**) and elemental maps (**right panels**; elements Ag, C, O and S were scanned) of the GSH-Ag NCs.

SEM and EDX analyses were used for the construction of elemental maps and the determination of atomic ratios for the GSH-Ag NCs (Figure 4). According to EDX data, the Ag:S atomic ratio measured was 1:0.65 ± 0.03. Based on the EDX and MALDI summary data, we proposed that the synthesized GSH-Ag NCs have putative Ag_35_(SG)_23_ composition.

**Figure 5:**
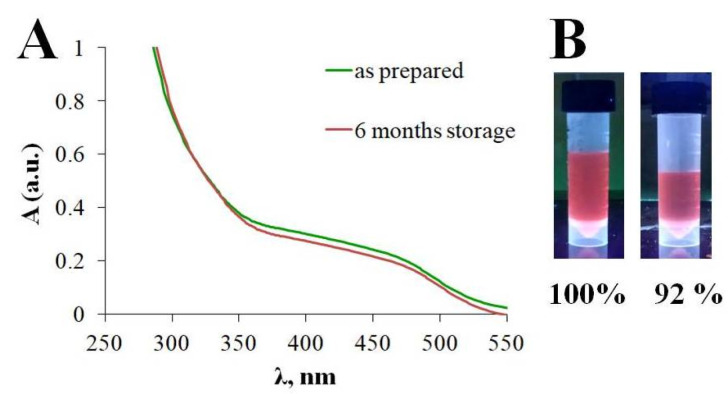
(**A**) UV−visible absorption spectra for the GSH-Ag NCs (**B**) Relative fluorescence intensity for “as prepared” GSH-Ag NCs and after six months of storage, respectively.

The stability of the synthesized GSH-Ag NCs was assessed after six months of storage using UV-Vis spectra and determination of fluorescence intensity (Figure 5). As shown in Figure 5A, extinction spectra of the GSH-Ag NCs have nosignifficant changes in the ultraviolet and visible regions. However, the relative fluorescence intensity of the GSH-Ag NCs was slightly decreased after six months of storage (Figure 5B). Thus, the synthesized GSH-Ag NCs were deemed to be quite stable, and NCs can be stored at 4 °C for at least six months.

### 2.2. Antibacterial Activity of the GSH-Ag NCs

At the first step, the antibacterial activity of glutathione-capped silver nanoclusters (GSH-Ag NCs) and silver nitrate was evaluated in vitro against planktonic bacterial cultures of Gram-positive strains (*S. aureus* 209P and *B. cereus* 8035) and Gram-negative bacteria (*E. coli* K-12 and *E. cloacae* K7) using the agar well-diffusion method (Table 1).

As shown in Table 1, a chelated silver of GSH-Ag nanoclusters demonstrated a significant antibacterial activity in vitro towards *S. aureus* 209P, *B. cereus* 8035, and *E. coli* K-12 bacterial strains, with a moderate effect observed in the case of *E. cloacae* K7. From the other side, free Ag^+^ ions (source AgNO_3_) showed only a moderate action towards *S. aureus* 209P, with a weak effect observed on the bacterial growth of *B. cereus* 8035, *E. coli* K-12, and *E. cloacae* K7 at the same concentration range, which is well correlated with previously published data [27].

A moderate/weak activity of Ag^+^ towards Gram-positive bacteria (*S. aureus* and *B. cereus*) seems to be logical and can be explained by the features of the bacterial cell wall of these pathogens. As previously known, Gram-positive bacteria have a much thicker layer of peptidoglycan in comparison with Gram-negative pathogens. The thicker cell wall of gram-positive bacteria has a crucial role in protecting the cell from the penetration of Ag^+^ ions into the cytoplasm [28]. Interestingly, the chelated silver of GSH-Ag NCs demonstrated a two-fold higher inhibitory potential against *S. aureus*, and a three-fold higher activity against *B. cereus* in comparison with free silver ions according to our experimental data. On an important note, ultra-small GSH-Ag NCs are significantly more active (about four times more) than glutathione-capped silver nanoparticles (~7 nm) against *S. aureus* [29].

**Figure 6:**
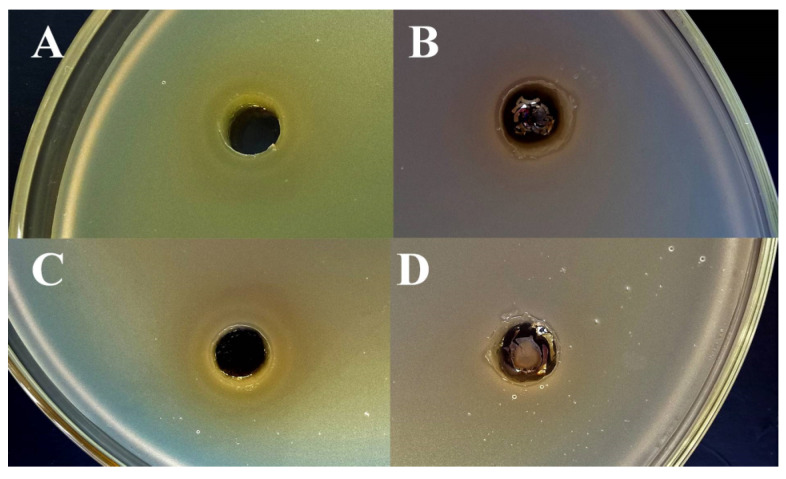
Agar well-diffusion assay for GSH-Ag NCs and Ag+. (**A**) Bacteriostatic activity of GSH-Ag NCs against *E. coli*. (**B**) Reduction of Ag+ ions by *E. coli*. (**C**) Bacteriostatic activity of GSH-Ag NCs against *E. cloacae*. (**D**) Reduction of Ag+ ions by *E. cloacae*.

In the case of Gram-negative bacteria (*E. coli* and *E. cloacae*), it was found that these types of bacteria have demonstrated an active in vitro reduction of Ag^+^ ions (AgNO_3_) to Ag^0^ (Figure 6B,D). For this reason, silver nitrate exhibits very low activity against *E. coli* and *E. cloacae* (Table 1). From the other side, all tested bacterial strains (including *E. coli* and *E. cloacae*) were crucially not able to reduce a chelated silver of glutathione-capped nanoclusters, which is a preserved antibacterial effect of GSH-Ag NCs, unlike the weak action of free Ag^+^ ions (Figure 6A,C). Therefore, GSH-Ag NCs were deemed to be demonstrate a strong inhibition against *E. coli* that is around three times more in comparison with the effects of AgNO_3_. Furthermore, GSH-Ag NCs displayed a notable bacteriostatic action in vitro on *E. cloacae* growth, unlike the very weak effect of AgNO_3_ against this bacterial strain (Table 1).

**Figure 7:**
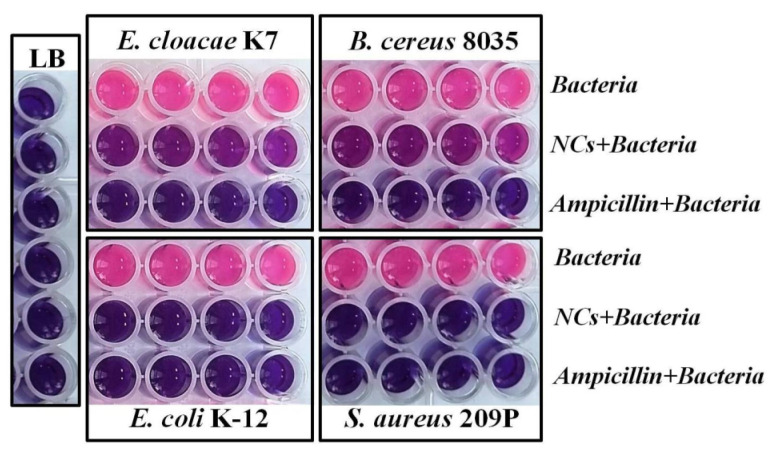
Resazurin metabolism assay for GSH-Ag NCs. Concentration of chelated silver of NCs: 84 μg/mL (*E. cloacae*); 42 μg/mL (*E. coli*); 56 μg/mL (*B. cereus*); and 42 μg/mL (*S. aureus*). Pink color indicates bacterial cell viability.

Next, a resazurin metabolism assay was performed to evaluateg the antibacterial effects of the GSH-Ag NCs on bacterial biofilm formation (Figure 7). Concentrations of silver nanoclusters and ampicillin (negative control) for each bacteria were chosen based on MIC data for planktonic cultures (see above, Table 1). As shown in Figure 7, GSH-Ag NCs have a strong potential to inhibit bacterial biofilm formation in the case of *S. aureus* and *E. coli*. From the other side, silver nanoclusters have demonstrated only a moderate effect on biofilm growth in the case of *B. cereus* and *E. cloacae*. Importantly, synthesized GSH-Ag NCs with putative Ag_35_(SG)_23_ composition display a stronger in vitro inhibition of *S. aureus* (about four times more) and *E. coli* (about one and half times) growth in comparison with the bioactivity of the previously described Ag_16_(SG)_9_ nanoclusters [30]. Probably, the antibacterial effect of Ag NCs depends on cluster composition. From the other side, surface modifications, such as the encapsulation of the GSH-Ag NCs in the phosphatidylcholine liposomes leads to an enhanced antibacterial activity (1.1–1.3 fold), higher stability, and biocompatibility in comparison with un-modified GSH-Ag NCs [19].

Moreover, we discovered that in vitro antibacterial activity of the synthesized GSH-Ag NCs against the copper-resistant bacterium *Achromobacter insolitus* LCu2 sharply increased (more than 100 times) in the presence of sub lethal concentration of Cu^2+^ in the culture medium (unpublished data). To explain the possible mechanisms of the antibacterial activity of silver nanoclusters, we performed a molecular docking of the GSH-Ag NCs with several bacterial targets (see below).

### 2.3. Molecular Docking of GSH-Ag NCs with Outer Membrane Protein TolC

Based on some our experimental data and data detailed in the literature [31], we selected several bacterial proteins (the outer membrane protein TolC, the cation efflux system protein CusF, and D-alanyl-D-alanine carboxypeptidase) as prospective targets for molecular docking with glutathione-capped silver nanoclusters.

The outer membrane bacterial protein TolC (PDB ID: 7NG9) is one of the most prevalent drug efflux channels in Gram-negative bacteria and is frequently associated with multidrug resistance. TolC family proteins are central to the export of small molecules, including metal ions and antibacterial drugs, which are harmful for bacteria [32]. Moreover, these proteins play a crucial role in the export of many large proteins, which include several proteins that aid bacterial survival in mammalian hosts [33]. Thus, the TolC protein is a possible chemotherapeutic target, which is important to the survival of pathogens during infections [34].

**Figure 8:**
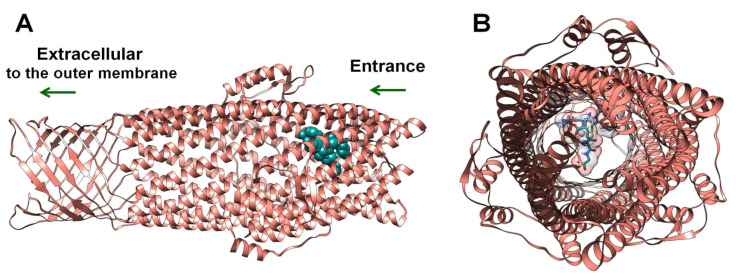
The docking pose of GSH-Ag NCs with the TolC protein. (**A**) Side view—depth of penetration of the GSH-Ag NCs into the periplasmic entrance of TolC. TolC depicted as ribbons (salmon), all atoms of the GSH-Ag NCs are presented in dark cyan (spheres). (**B**) View from entrance of TolC—TolC depicted as ribbons (salmon), all atoms of the GSH-Ag NCs are shown as sticks (carbon atoms in dark cyan, and silver atoms as silver spheres). VdW surface of the GSH-Ag NCs have demonstrated a partial occupation of the periplasmic entrance of TolC.

According to Autodock data, glutathione-capped silver nanoclusters have quite a significant affinity to the outer membrane protein TolC (Table 2). Integrally, GSH-Ag NCs were accommodated into the periplasmic entrance of bacterial TolC by docking studies (Figure 8). Furthermore, GSH-Ag NCs displayed many interactions with the amino acid residues of the conserved entrance aperture of TolC (Table 2, Figure S2). Moreover, silver nanoclusters also demonstrated Van der Waals interactions (VdW) with Thr122, Ala163, Asn167, Thr371, and Thr372. Based on these data, we propose that binding GSH-Ag NCs with TolC can disrupt the functions of this bacterial efflux pump (with the removal of antibiotics and metal ions from the bacterial cell) by stopping a crucial transition of TolC from closed-state to open-state [35].

### 2.4. Molecular Docking of the GSH-Ag NCs with the Cation Efflux System Protein CusF

As revealed from previous research, bacterial cells have a genetically determined resistance to toxic and nonessential metal ions, such as Ag^+^, Cd^2+^, Hg^2+^, and Pb^2+^ [36]. Bacterial silver resistance has been reported earlier [37,38,39] and, for example, in a random collection of enteric bacteria from a Chicago hospital, where more than 10% had genes for Ag+ resistance [40]. Ag^+^-resistant bacteria were shown to have an active Ag+ efflux pump, such as the CusCBFA system [41]. The cation efflux system protein CusF (PDB ID: 1ZEQ) is a periplasmic Ag+/Cu+-binding protein that probably functions as a chaperone to carry Ag+ or Cu+ from the bacterial cell to the equivalent CusCBFA efflux pump. Thus, the bacterial metal-binding protein CusF is a possible target for drug discovery, which is involved in Ag+ resistance [42].

**Figure 9:**
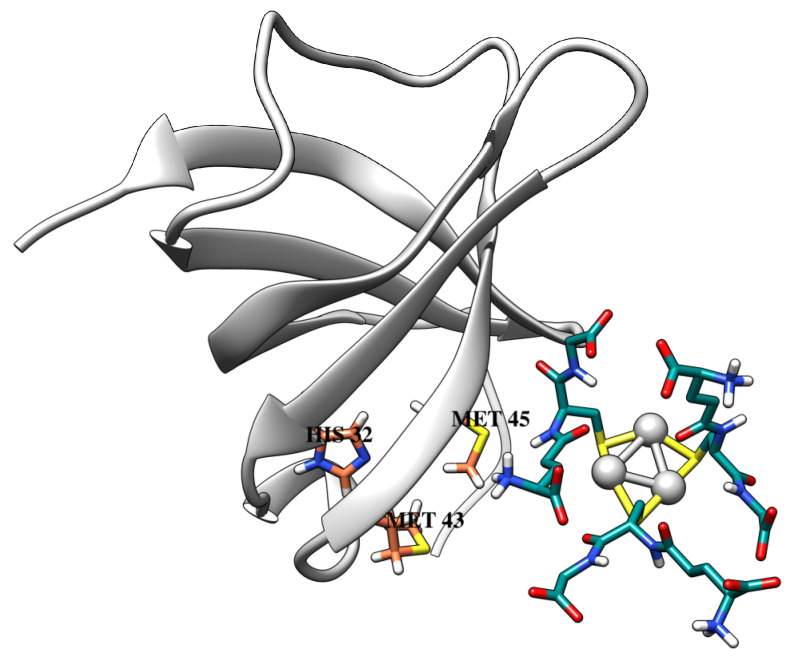
The docking pose of GSH-Ag NCs with the CusF protein. CusF depicted as ribbons (light gray), and metal-binding active site residues are shown as sticks (carbon atoms in coral). All atoms of the GSH-Ag NCs are shown as sticks (carbon atoms in dark cyan, and silver atoms as silver spheres).

From docking data, silver nanoclusters were found to have a moderate affinity to the metal-binding protein CusF (Table 2). Importantly, GSH-Ag NCs were accommodated nearby with the active site of CusF (His32, Met43, and Met45 main residues) by in silico results (Figure 9). These residues are forming the metal–ion binding site, which is important for CusF protein function [43]. Moreover, GSH-Ag NCs have demonstrated different interactions with the active site residues of the CusF protein (Table 2, Figure S3). Silver nanoclusters also displayed VdW interactions with Met43, Thr48, Asn73, and Leu74 residues. Therefore, as according to Autodock data, GSH-Ag NCs have a favorable binding with the metal-binding protein CusF, by forming strong hydrogen bonds, and other interaction types with the amino acid residues of CusF. Based on the docking results, this type of silver nanocluster is a possible inhibitor of the bacterial protein CusF. Thus, obtained in silico data are important for the discovery of antimicrobial agents against clinical Ag^+^-resistant bacterial strains.

### 2.5. Molecular Docking of the GSH-Ag NCs with D-Alanyl-D-Alanine Carboxypeptidase

Almost all bacteria have a cross-linked cell wall, which imparts structural strength and shape to bacteria. The cross-linking reaction of peptidoglycan chains is the last step in cell wall biosynthesis [44]. D-alanyl-D-alanine carboxypeptidase (DD-peptidase, PDB ID: 1MPL) is a β-lactam-sensitive enzyme, which is responsible for the final and critical peptidoglycan cross-linking step in bacterial cell wall biosynthesis. For this reason, inhibition of DD-peptidase disrupts the process of cell wall synthesis that is crucial for bacterial survival [45].

**Figure 10:**
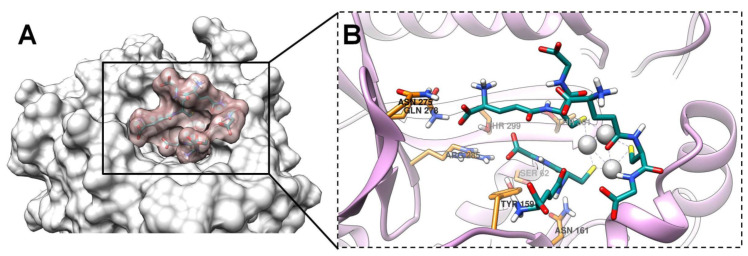
The docking pose of GSH-Ag NCs with DD-peptidase. (**A**) The occupation of the hydrophilic pocket in the active site of protein. The VdW surface of DD-peptidase is presented in light gray, and the VdW surface of GSH-Ag NCs is shown in rosy brown. (**B**) Magnified view (with focus on the ligand)—accommodation of the GSH-Ag NCs within the active site of DD-peptidase. DD-peptidase is depicted as ribbons (plum), and the active site residues are shown as sticks (carbon atoms in orange). All atoms of the GSH-Ag NCs are shown as sticks (carbon atoms in dark cyan and silver atoms as silver spheres).

Glutathione-capped silver nanoclusters have demonstrated a moderate affinity to DD-peptidase, according to Autodock data (Table 2). From examining the results of the docking studies, GSH-Ag NCs were found to be well accommodated within the active site of DD-peptidase (Figure 10B). Furthermore, GSH-Ag NCs displayed a sufficient occupation of the hydrophilic pocket in the active site of the target (Figure 10A). Moreover, silver nanoclusters were found to have VdW interactions with Tyr159, Ser160, Asn161, Asn275, and Arg285 residues of the active site of DD-peptidase (Figure S4). GSH-Ag NCs also displayed strong hydrogen bonds with the Asp114 and Ser158 residues of this protein (Table 2). Therefore, GSH-Ag NCs might be considered as a possible inhibitor of DD-peptidase with moderate potential according to docking data.

Molecular docking was performed to explain the possible mechanisms of the GSH-Ag NCs actions on bacterial survival and multidrug resistance. Crucially, GSH-Ag NCs were found to have demonstrated a moderate affinity to several bacterial proteins based on the docking data. Thus, GSH-Ag NCs possibly respond to requirements of a multi-target methodology in the drug design, which implies the partial inhibition of several targets simultaneously by one compound. In fact, surprising advances in multi-target drug discovery have demonstrated that the partial inhibition of a small number of targets can be more effective than the full inhibition of only one target [46].

### 2.6. In Silico Prediction of Pharmacokinetic and Toxicity Properties for GSH-Ag NCs

Pharmacokinetic and toxicity properties (ADMET) of molecules are one of the main parameters for drug discovery. Clinical trials often detect undesirable toxicity and unsatisfactory pharmacokinetics (poor absorption, high level of primary metabolism, etc.) for drug candidate molecules. For this reason, prediction of ADMET profiles in silico is a useful tool for the rapid and preliminary evaluation of pharmacokinetic and toxicity properties. Thus, the ADMET profile of GSH-Ag NCs was predicted using the pkCSM platform (Table 3).

Importantly, GSH-Ag NCs have no potential as substrates and inhibitors of the P-glycoprotein, according to data in silico. The P-glycoprotein acts as a biological barrier and transporter for removing toxins and xenobiotics out of the cells [47]. Distribution of silver nanoclusters was assessed in silico using BBB permeability (logarithm of permeability in blood–brain barrier) and CNS permeability (blood–brain permeability-surface-area product) parameters. Any molecule is able to cross the blood–brain barrier if the predicted value of logBB > 0.3. In addition, compounds with a logPS > −2 are considered to penetrate the CNS [48]. Thus, GSH-Ag NCs are unable to penetrate the CNS (Table 3).

The metabolism and excretion of silver nanoclusters were evaluated by prediction cytochrome P-450 isoforms (CYP) substrate and inhibitory potential, and for potential as substrate of renal organic cation transporter 2 (OCT2). Cytochrome P-450 is a crucial detoxification enzyme in the body, and many drugs are inactivated by CYP. Organic cation transporter 2 is a renal uptake transporter that plays an important role in the desposition and renal clearance of drugs and endogenous compounds [49]. As shown in Table 3, silver nanoclusters practically have no problems with metabolism and excretion from an organism.

Undesirable toxic effects of compounds are one of the main problems in drug development. Such important parameters as AMES toxicity, inhibition of human ERG channels, hepatotoxicity, and skin sensitization were predicted for GSH-Ag NCs with the pkCSM platform. AMES toxicity is a widely employed method to assess the mutagenic potential of ligands using bacteria [50]. Inhibition of the potassium channels encoded by hERG will induce a potential cardiac toxicity of molecules. Drug-induced liver injury is a major safety concern for drug discovery [49]. According to the pkCSM data, GSH-Ag NCs have no potential mutagenicity, cardiac toxicity, and hepatotoxicity (Table 3). Therefore, according to the predictive data, glutathione-capped silver nanoclusters have an excellent ADMET profile and may be used for further studies in vivo.

### 2.7. Future Directions for Research of Silver Nanoclusters

Ultra-small silver nanoclusters are promising antibacterial agents, which have displayed a higher inhibitory potential to Gram-positive and Gram-negative bacteria in comparison with free silver ions. However, only a deep understanding of the underlying molecular mechanisms of action may lead to the successful design of silver-based, next-generation antibiotics against multi-drug resistant pathogens. Moreover, it is urgently needed to perform a comprehensive in vitro and in vivo evaluation of Ag NC toxicity for potential applications in medicine. Furthermore, the stability of Ag nanoclusters may be insufficient for in vivo application. Importantly, the chemical nature of the capping ligand (in particular, the charge of the NC surface) is crucial for the bioactivity and stability of nanoclusters. From the other side, the surface modification of Ag NCs (encapsulation, etc.) seems to be a prospective method for increasing the stability and biocompatibility of Ag NCs, despite the difficulties of this approach.

## 3. Materials and Methods

### 3.1. Materials

Silver nitrate (AgNO_3_, ≥99.0%), L-glutathione reduced (GSH, ≥98.0%), sodium hydroxide (NaOH, ≥98.0%), sodium borohydride (NaBH_4_, 99%), ethanol (EtOH, ≥99.5%), and ampicillin sodium salt were all purchased from Sigma-Aldrich (Saint Louis, MO, USA). Ultrapure water with a resistivity of 18.2 MΩ × cm (Milli-Q Integral 5 system, Merck Millipore, Billerica, MA, USA) was uitlized in all experiments.

### 3.2. Synthesis of GSH-Ag NCs

The synthesis of GSH-Ag NCs was previously reported with modifications [51]. Briefly, the fresh solutions of AgNO_3_ (500 μL, 25 mM) and GSH (500 μL, 25 mM) were added into ultrapure water (4 mL) at room temperature, and reacted under magnetic stirring of 500 rpm to form GSH-Ag(I) complex as a white precipitate. Subsequently, NaOH solution (25 μL, 1 M) was added and a colorless solution was formed. Next, NaBH_4_ solution (48 μL, 260 mM) was added dropwise to the solution under vigorous stirring at room temperature. After 1 h, the reaction mixture was placed in a refrigerator at 4 °C for 12 h to allow slow etching. Finally, the synthesized GSH-Ag NCs were purified using an Amicon Ultra-15 centrifugal filter with a molecular weight cutoff of 3 kDa (Merck Millipore, Billerica, MA, USA), and then re-dissolved in ultrapure water. The resultant GSH-Ag NC solution was stored in a refrigerator at 4 °C for further use.

### 3.3. Characterization of GSH-Ag NCs

Optical UV-Vis extinction spectra of the GSH-Ag NCs were recorded by a Specord S600 (Analytic Jena, Jena, Germany) in a range of 250–800 nm. Fluorescent measurements were performed using the Cary Eclipse fluorescence spectrophotometer (Agilent, Santa Clara, CA, USA). The transmission electron microscopy (TEM) observations of the GSH-Ag NCs were performed using the Libra-120 electron microscope (Carl Zeiss, Oberkochen, Germany) at the the Simbioz Center for the Collective Use of Research Equipment in the Field of Physical-Chemical Biology and Nanobiotechnology, IBPPM RAS, Saratov. The hydrodynamic diameter (DLS) of the GSH-Ag NCs was measured using a Zetasizer Nano ZS (Malvern, UK).

### 3.4. Thin-Layer Chromatography (TLC) Analysis

The sample of GSH-Ag NCs (1 μL) was spotted to a TLC plate (chromatographic paper FN100, 195 g/m^2^, Munktell, Dia-M) and dried in air. After drying, the plate was eluted in the descending variant of TLC with the H_2_O/EtOH/1M NaOH (200/40/1, *v*/*v*/*v*) solvent system. After the separation and drying stages, GSH-Ag NCs were detected (with the naked-eye and under UV illumination at 312 nm), and the retention factor (Rf) of the NCs was calculated.

### 3.5. MALDI-MS

MALDI mass spectra were recorded on the Axima Confidence time-of-flight spectrometer (Shimadzu Biotech, Columbia, MD, USA) in the linear mode with the nitrogen laser (λ = 337.1 nm; maximum firing rate, 60 Hz; maximum power, 150 μJ/pulse). Positive ions were recorded and the mass spectra were averaged for approximately 100 shots. The mass range of *m*/*z* 500–20,000 was scanned. CHCA (α-cyano-4-hydroxycinnamic acid) was used as the matrix for all measurements. The matrix solution was prepared by dissolving 1 mg of CHCA in 100 μL of a 1:1 (*v*/*v*) mixture of acetonitrile and water with 0.1% trifluoroacetic acid (TFA). On the MALDI target plate, 1 μL of sample followed by 2 μL of matrix solution was deposited and dried in air.

### 3.6. SEM/EDX

Scanning electron microscopic (SEM, MIRA 2 LMU, Tescan, Brno, Czech Republic) and energy dispersive X-ray (EDX, AztecLive Advanced Ultim Max 40) analyses were performed in the Laboratory for Diagnostics of Nanomaterials and Structures of the Educational and Scientific Institute of Nanostructures and Biosystems of Saratov State University. For measurements, samples of the GSH-Ag NCs were dropped on silicon wafer and dried in vacuum.

### 3.7. Bacterial Cultures

We used the Gram-positive *Staphylococcus aureus* 209-P and *Bacillus cereus* 8035, as well as the Gram-negative *Escherichia coli* K-12 and *Enterobacter cloacae* K7 in this work. Bacterial cultures were grown in Luria-Bertani (LB) medium (10 g/L tryptone, 5 g/L yeast extract, 5 g/L NaCl) at 37 °C.

### 3.8. Agar-Well Diffusion Method

The antimicrobial activity of the GSH-Ag NCs toward planktonic bacterial cultures was measured using the agar-well diffusion method. Overnight bacterial cultures were diluted to 1 × 10^8^ CFU/mL (2 mL) and mixed with preheated LB medium with 2% agar (25 mL) followed by plating on sterile petri dishes. After the gelation of the agar medium, wells with a diameter of 5 mm were cut in each agar plate with a sterilized metallic borer. Next, 200 µL of compounds were added to the respective wells. The standard antibacterial drug ampicillin was used as the positive control. After adding the relevant compounds, bacterial cultures were incubated for 24 h at 37 °C. The antibacterial activity was determined by measuring the average of the three readings diameter of zones showing complete inhibition.

### 3.9. Biofilm Formation and Resazurin Metabolism Assay

Resazurin assay was performed for the evaluation of the antibacterial properties of the GSH-Ag NCs towards the bacterial biofilms of *Staphylococcus aureus* 209-P, *Bacillus cereus* 8035, *Escherichia coli* K-12, and *Enterobacter cloacae* K7. Overnight bacterial cultures were diluted to 1 × 10^5^ CFU/mL in sterile LB medium. Next, 200 µL of diluted cultures were transferred to a new 96-well microplate. Aliquots of GSH-Ag NCs were added to the appropriate wells with the final concentration of the NCs (42–84 µg/mL on the basis of Ag atoms) depending on bacterial strain. LB medium and ampicillin were used as the positive and negative growth controls, respectively. After adding the nanoclusters, bacterial cultures were then incubated for 72 h at 37 °C. Following this, a solution of 0.01 % (*w*/*v*) resazurin in water (200 µL) was added to each well, and the bacterial cells were incubated for 1 h at 37 °C until the mixture produced a color. Pink color is indicative of bacterial cell viability.

### 3.10. Molecular Docking

To perform the molecular docking of GSH-Ag NCs with bacterial targets, we selected the Ag_3_(SG)_3_ structure as the input ligand, due to the unavailability of the crystal structure for putative Ag_35_(SG)_23_ cluster composition, and the limitation of the number of rotatable bonds for the ligands in AutoDock 4.2.6.

The initial structure of Ag_3_(SG)_3_ was built up with the help of Avogadro 1.2.0 [52] by replacing the MBA ligand using a model of 2-mercaptobenzoic acid-capped silver nanoclusters Ag_3_(MBA)_3_ taken from its known crystal structure [53]. The 3D structure of Ag_3_(SG)_3_ was optimized with the universal force field (UFF) in keeping the Ag and S atoms fixed with a steepest descent algorithm (500 steps) using Avogadro 1.2.0.

Experimental bacterial protein structures of the outer membrane channel protein TolC (PDB ID: 7NG9, method—electron microscopy, resolution 3.30 Å), the cation efflux system protein CusF (PDB ID: 1ZEQ, method—X-ray diffraction, resolution 1.50 Å), and D-alanyl-D-alanine carboxypeptidase (PDB ID: 1MPL, method—X-ray diffraction, resolution 1.12 Å) were downloaded from the protein data bank (PDB). MolProbity was used to assess the quality of the structural models of the target proteins, assign protonation states, add missing hydrogen atoms, and optimize hydrogen-bonding networks [54]. Prior to molecular docking, all the co-crystallized ligands and the solvent molecules were removed from the experimental structural models of proteins. A molecular blind docking method with a Lamarckian genetic algorithm was used to study the interactions of the GSH-Ag NCs with their bacterial targets using AutoDock 4.2.6 [55]. Parameters of the silver atoms were additionally added for molecular docking. Kollman charges were added for proteins, and Gasteiger charges were applied for the ligand. The following docking parameters were used: for TolC protein (PDB ID: 7NG9)—number of XYZ grid points of cubic simulation cell (100 × 100 × 126 Å), spacing (1.0 Å), XYZ coordinates of the grid center (88.797, 89.314, 112.741); for CusF protein (PDB ID: 1ZEQ)—number of XYZ grid points of cubic simulation cell (80 × 80 × 80 Å), spacing (1.0 Å), XYZ coordinates of the grid center (−2.987, 9.07, 3.85); and for DD-peptidase (PDB ID: 1MPL)—number of XYZ grid points of cubic simulation cell (80 × 80 × 80 Å), spacing (1.0 Å), XYZ coordinates of the grid center (13.93, −6.87, 36.04).

Other parameters were set to default values. Input ligand with polar hydrogens was kept as rigid and used in a pdbqt format for docking. Three-dimensional images of the docking results were generated using UCSF Chimera 1.14 [48]. Important protein-ligand interactions were detected using the BIOVIA Discovery Studio^®^ Visualizer 21.1.0.20298 [56].

### 3.11. In Silico ADMET Profile of GSH-Ag NCs

In silico pharmacokinetic and toxicity parameters (absorption, distribution, metabolism, excretion, and toxicity [ADMET] profile) such as P-glycoprotein substrate and inhibitory potential, BBB permeability (logarithm of permeability in blood–brain barrier), CNS permeability (blood–brain permeability–surface area product), potential as substrate of renal organic cation transporter 2 (OCT2), AMES toxicity, inhibition of human ERG channels, hepatotoxicity, and skin sensitization were all predicted using the pkCSM platform [57].

## 4. Conclusions

In summary, we have developed a simple synthesis of novel fluorescent GSH-Ag NCs with enhanced antibacterial activity. According to the TLC analysis data, the synthesized GSH-Ag NCs are individual fractions with one cluster composition without any impurities. Based on the data of the MALDI-MS and EDX methods, the fabricated silver nanoclusters have putative Ag_35_(SG)_23_ composition with molecular weight of about 11 kDa. We have shown that the chelated silver of GSH-Ag NCs has a higher antibacterial activity in comparison with the free Ag+ ions of AgNO_3_ towards both Gram-positive and Gram-negative bacteria. Moreover, according to molecular docking, GSH-Ag NCs have demonstrated a moderate inhibitory potential to three bacterial proteins: the outer membrane protein TolC, the cation efflux system protein CusF, and D-alanyl-D-alanine carboxypeptidase, which are all crucial for bacterial survival and multidrug resistance. Furthermore, GSH-Ag NCs have an excellent ADMET profile in silico without undesirable toxic effects and may therefore be used for further investigations in vivo.

## Figures and Tables

**Table 1:** The antibacterial activity of the GSH-Ag NCs in comparison with Ag+ ions.

Compound	Minimal Inhibitory Concentration (MIC, μg/mL)			
	*Staphylococcus aureus* 209P	*Bacillus cereus* 8035	*Escherichia coli* K-12	*Enterobacter cloacae* K7
* GSH-Ag NCs	42	56	42	84
* AgNO_3_	86	168	134	>168
L-glutathione	>500	N.D.	>500	N.D.
Ampicillin	2	10	4	4

* MIC was detected on the basis of Ag atoms. N.D.—not determined.

**Table 2:** Autodock ∆G values and key interactions for the best docked complexes of the GSH-Ag NCs with bacterial targets.

Bacterial Target	∆G_intermol_ ^a^, kcal/mol	∆G_elec_ ^b^, kcal/mol	Interacting Residues (Type of Interaction and Distance in Å)
Outer membrane protein TolC (PDB ID: 7NG9)	−5.94	+0.96	Gln160 (HB, 2.84), Thr170 (carbon HB ^c^, 3.48), Tyr338 (HB, 2.13), Thr372 (HB, 3.14, 3.21), Asn375 (HB, 2.51, 2.85), Gln378 (HB, 2.53), Gln379 (unfavorable donor-donor, 5.59)
Metal-binding protein CusF (PDB ID: 1ZEQ)	−5.07	−2.32	Lys27 (HB, 2.37), Thr44 (HB, 2.50, 2.79), Met45 (alkyl, 3.54), Arg46 (HB, 2.13, 2.48, charge repulsion, 5.43), Ser75 (HB, 2.90)
D-alanyl-D-alanine carboxypeptidase (PDB ID: 1MPL)	−4.84	−0.41	Asp114 (HB, 2.35), Asn117 (metal-acceptor, 3.06), Ser158 (HB, 1.90)

^a^ Final intermolecular energy is a sum of VdW dispersion/repulsion, hydrogen bonding, electrostatics, and desolvation energies ∆G_intermol_ = ∆G_VdW_ + ∆G_hbond +_ ∆G_elec_ + ∆G_desolv_. ^b^ Energy of electrostatic interactions. ^c^ HB—Conventional hydrogen bond.

**Table 3:** In silico ADMET pharmacokinetic profile of the GSH-Ag NCs.

Property	Model	Predicted Value
Absorption	P-glycoprotein substrate	No
	P-glycoprotein I inhibitor	No
	P-glycoprotein II inhibitor	No
Distribution	BBB permeability	−2.1
	CNS permeability	−5.8
Metabolism	CYP2D6 substrate	No
	CYP3A4 substrate	Yes
	CYP1A2 inhibitor	No
	CYP2C19 inhibitor	No
	CYP2C9 inhibitor	No
	CYP2D6 inhibitor	No
	CYP3A4 inhibitor	No
Excretion	Renal OCT2 substrate	No
Toxicity	AMES toxicity	No
	hERG I inhibitor	No
	hERG II inhibitor	No
	Hepatotoxicity	No
	Skin sensitization	No

## Data Availability

Data can be shared upon request.

## Supplementary Materials

The following supporting information can be downloaded at: https://www.mdpi.com/article/10.3390/ijms24098306/s1.

## References

[1] Chernousova S., Epple M. (2013). Silver as antibacterial agent: Ion, nanoparticle, and metal. Angew. Chem. Int. Ed..

[2] Klasen H. (2000). Historical review of the use of silver in the treatment of burns. I. Early Uses Burns.

[3] Slawson R.M., Van Dyke M.I., Lee H., Trevors J.T. (1992). Germanium and silver resistance, accumulation, and toxicity in microorganisms. Plasmid.

[4] Marambio-Jones C., Hoek E.M.V. (2010). A review of the antibacterial effects of silver nanomaterials and potential implications for human health and the environment. J. Nanopart. Res..

[5] Dror-Ehre A., Mamane H., Belenkova T., Markovich G., Adin A. (2009). Silver nanoparticle–*E. coli* colloidal interaction in water and effect on E. coli survival. J. Colloid Interface Sci..

[6] Thurman R.B., Gerba C.P., Bitton G. (1989). The molecular mechanisms of copper and silver ion disinfection of bacteria and viruses. Crit. Rev. Environ. Control.

[7] Jung W.K., Koo H.C., Kim K.W., Shin S., Kim S.H., Park Y.H. (2008). Antibacterial activity and mechanism of action of the silver ion in *Staphylococcus aureus* and *Escherichia coli*. Appl. Environ. Microbiol..

[8] Arora S., Jain J.M., Rajwade J., Paknikar K.M. (2008). Cellular responses induced by silver nanoparticles: In vitro studies. Toxicol. Lett..

[9] Zheng K., Setyawati M.I., Leong D.T., Xie J. (2018). Antimicrobial silver nanomaterials. Coord. Chem. Rev..

[10] Akavaram S., Desai M.L., Park T.-J., Murthy Z., Kailasa S.K. (2020). Trypsin encapsulated gold-silver bimetallic nanoclusters for recognition of quinalphos via fluorescence quenching and of Zn^2+^ and Cd^2+^ ions via fluorescence enhancement. J. Mol. Liq..

[11] Desai M.L., Jha S., Basu H., Singhal R.K., Sharma P., Kailasa S.K. (2018). Chicken egg white and L-cysteine as cooperative ligands for effective encapsulation of Zn-doped silver nanoclusters for sensing and imaging applications. Colloids Surf. A Physicochem. Eng. Asp..

[12] Jiang X., Du B., Huang Y., Zheng J. (2018). Ultrasmall noble metal nanoparticles: Breakthroughs and biomedical implications. Nano Today.

[13] Buceta D., Busto N., Barone G., Leal J.M., Domínguez F., Giovanetti L.J., López-Quintela M.A. (2015). Ag_2_ and Ag_3_ clusters: Synthesis, characterization, and interaction with DNA. Angew. Chem..

[14] Neissa J., Pérez-Arnaiz C., Porto V., Busto N., Borrajo E., Leal J.M., Dominguez F. (2015). Interaction of silver atomic quantum clusters with living organisms: Bactericidal effect of Ag_3_ clusters mediated by disruption of topoisomerase–DNA complexes. Chem. Sci..

[15] Wu J., Hou S., Ren D., Mather P.T. (2009). Antimicrobial properties of nanostructured hydrogel webs containing silver. Biomacromolecules.

[16] Batarseh K.I. (2004). Anomaly and correlation of killing in the therapeutic properties of silver (I) chelation with glutamic and tartaric acids. J. Antimicrob. Chemother..

[17] Elokely K., Doerksen R.J. (2013). Docking challenge: Protein sampling and molecular docking performance. J. Chem. Inf. Model..

[18] Torres P.H., Sodero A.C., Jofily P., Silva F.P. (2019). Key topics in molecular docking for drug design. Int. J. Mol. Sci..

[19] Liu J., Li X., Liu L., Bai Q., Sui N., Zhu Z. (2021). Self-assembled ultrasmall silver nanoclusters on liposome for topical antimicrobial delivery. Colloids Surf. B Biointerfaces.

[20] Díez I., Ras R.H. (2011). Fluorescent silver nanoclusters. Nanoscale.

[21] Rurack K., Spieles M. (2011). Fluorescence quantum yields of a series of red and near-infrared dyes emitting at 600−1000 nm. Anal. Chem..

[22] Udayabhaskararao T., Bootharaju M.S., Pradeep T. (2013). Thiolate-protected Ag32 clusters: Mass spectral studies of composition and insights into the Ag-thiolate structure from NMR. Nanoscale.

[23] Ghosh A., Hassinen J., Pulkkinen P., Tenhu H., Ras R.H.A., Pradeep T. (2014). Simple and efficient separation of atomically precise noble metal clusters. Anal. Chem..

[24] Tian S., Li Y.-Z., Li M.-B., Yuan J., Yang J., Wu Z., Jin R. (2015). Structural isomerism in gold nanoparticles revealed by X-ray crystallography. Nat. Commun..

[25] Yan N., Xia N., Liao L., Zhu M., Jin F., Jin R., Wu Z. (2018). Unraveling the long-pursued Au_144_ structure by X-ray crystallography. Sci. Adv..

[26] Chaki N.K., Negishi Y., Tsunoyama H., Shichibu Y., Tsukuda T. (2008). Ubiquitous 8 and 29 kDa Gold: Alkanethiolate cluster compounds: Mass-spectrometric determination of molecular formulas and structural implications. J. Am. Chem. Soc..

[27] Salman H.D. (2017). Evaluation and comparison the antibacterial activity of silver nano particles (AgNPs) and silver nitrate (AgNO_3_) on some pathogenic bacteria. J. Glob. Pharma. Technol..

[28] Feng Q.L., Wu J., Chen G.Q., Cui F.Z., Kim T.N., Kim J.O. (2000). A mechanistic study of the antibacterial effect of silver ions on Escherichia coli and Staphylococcus aureus. J. Biomed. Mater. Res..

[29] Amato E., Diaz-Fernandez Y.A., Taglietti A., Pallavicini P., Pasotti L., Cucca L., Milanese C., Grisoli P., Dacarro C., Fernandez-Hechavarria J.M. (2011). Synthesis, characterization and antibacterial activity against gram positive and gram negative bacteria of biomimetically coated silver nanoparticles. Langmuir.

[30] Wang Z., Fang Y., Zhou X., Li Z., Zhu H., Du F., Yuan X., Yao Q., Xie J. (2020). Embedding ultrasmall Ag nanoclusters in Luria-Bertani extract via light irradiation for enhanced antibacterial activity. Nano Res..

[31] Srivastava S., Bhargava A., Pathak N., Srivastava P. (2019). Production, characterization and antibacterial activity of silver nano-particles produced by Fusarium oxysporum and monitoring of protein-ligand interaction through in-silico approaches. Microb. Pathog..

[32] Poole K. (2002). Outer membranes and efflux: The path to multidrug resistance in gram-negative bacteria. Curr. Pharm. Biotechnol..

[33] Paulsen I.T., Park J.H., Choi P.S., Saier M.H. (1997). A family of gram-negative bacterial outer membrane factors that function in the export of proteins, carbohydrates, drugs and heavy metals from gram-negative bacteria. FEMS Microbiol. Lett..

[34] Koronakis V., Eswaran J., Hughes C. (2004). Structure and function of TolC: The bacterial exit duct for proteins and drugs. Annu. Rev. Biochem..

[35] Budiardjo S.J., Stevens J.J., Calkins A.L., Ikujuni A.P., Wimalasena V.K., Firlar E., Slusky J.S. (2022). Colicin E1 opens its hinge to plug TolC. Elife.

[36] Silver S., Phung L.T. (2005). A bacterial view of the periodic table: Genes and proteins for toxic inorganic ions. J. Ind. Microbiol. Biotechnol..

[37] Davis I.J., Richards H., Mullany P. (2005). Isolation of silver- and antibiotic-resistant *Enterobacter cloacae* from teeth. Oral Microbiol. Immunol..

[38] Li X.Z., Nikaido H., Williams K.E. (1997). Silver-resistant mutants of Escherichia coli display active efflux of Ag^+^ and are deficient in porins. J. Bacteriol..

[39] Mchugh G.L., Moellering R., Hopkins C., Swartz M. (1975). *Salmonella typhimurium* resistant to silver nitrate, chloramphenicol, and ampicillin: A new threat in burn units?. Lancet.

[40] Silver S. (2003). Bacterial silver resistance: Molecular biology and uses and misuses of silver compounds. FEMS Microbiol. Rev..

[41] Franke S., Grass G., Nies D.H. (2001). The product of the ybdE gene of the Escherichia coli chromosome is involved in detoxification of silver ions. Microbiology.

[42] Franke S., Grass G., Rensing C., Nies D.H. (2003). Molecular analysis of the copper-transporting efflux system CusCFBA of *Escherichia coli*. J. Bacteriol..

[43] Loftin I.R., Franke S., Roberts S.A., Weichsel A., Héroux A., Montfort W.R., Rensing C., McEvoy M.M. (2005). A novel copper-binding fold for the periplasmic copper resistance protein CusF. Biochemistry.

[44] Lee W., McDonough M.A., Kotra L.P., Li Z.-H., Silvaggi N.R., Takeda Y., Kelly J.A., Mobashery S. (2001). A 1.2-Å snapshot of the final step of bacterial cell wall biosynthesis. Proc. Natl. Acad. Sci. USA.

[45] Silvaggi N.R., Anderson J.W., Brinsmade S.R., Pratt R.F., Kelly J.A. (2003). The crystal structure of phosphonate-inhibited d-Ala-d-Ala peptidase reveals an analogue of a tetrahedral transition state. Biochemistry.

[46] Csermely P., Agoston V., Pongor S. (2005). The efficiency of multi-target drugs: The network approach might help drug design. Trends Pharmacol. Sci..

[47] Prachayasittikul V., Prachayasittikul V. (2016). P-glycoprotein transporter in drug development. EXCLI J..

[48] Pettersen E.F., Goddard T.D., Huang C.C., Couch G.S., Greenblatt D.M., Meng E.C., Ferrin T.E. (2004). UCSF Chimera? A visualization system for exploratory research and analysis. J. Comput. Chem..

[49] Harpole T.J., Delemotte L. (2018). Conformational landscapes of membrane proteins delineated by enhanced sampling molecular dynamics simulations. Biochim. Biophys. Acta.

[50] Benigni R., Bossa C. (2018). Data-based review of QSARs for predicting genotoxicity: The state of the art. Mutagenesis.

[51] Liu J., Li S., Fang Y., Zhu Z. (2019). Boosting antibacterial activity with mesoporous silica nanoparticles supported silver nanoclusters. J. Colloid Interface Sci..

[52] Hanwell M.D., Curtis D.E., Lonie D.C., Vandermeersch T., Zurek E., Hutchison G.R. (2012). Avogadro: An advanced semantic chemical editor, visualization, and analysis platform. J. Cheminf..

[53] Bi Y., Wang Z., Liu T., Sun D., Godbert N., Li H., Hao J., Xin X. (2021). Supramolecular chirality from hierarchical self-assembly of atomically precise silver nanoclusters induced by secondary metal coordination. ACS Nano.

[54] Williams C.J., Headd J.J., Moriarty N.W., Prisant M.G., Videau L.L., Deis L.N., Verma V., Keedy D.A., Hintze B.J., Chen V.B. (2018). MolProbity: More and better reference data for improved all-atom structure validation. Protein Sci..

[55] Morris G.M., Huey R., Lindstrom W., Sanner M.F., Belew R.K., Goodsell D.S., Olson A.J. (2009). AutoDock4 and AutoDockTools4: Automated docking with selective receptor flexibility. J. Comput. Chem..

[56] BIOVIA (2022). Dassault Systèmes.

[57] Pires D.E.V., Blundell T.L., Ascher D.B. (2015). pkCSM: Predicting small-molecule pharmacokinetic and toxicity properties using graph-based signatures. J. Med. Chem..

