# Supplementary material for: Enhanced Antibacterial Activity of Novel Fluorescent Glutathione-Capped Ag Nanoclusters
Source: Int J Mol Sci. 2023 May 5;24(9):8306. doi: 10.3390/ijms24098306 (PMC10179335; doi:10.3390/ijms24098306)
Supplement: Supplementary file 1 [file ijms-24-08306-s001.zip › ijms-2357809-supplementary.pdf]

Synthesis of novel fluorescent glutathione-capped silver nanoclusters with enhanced antibacterial activity

*Corresponding author: khlebtsov_b@ibppm.ru (Boris N. Khlebtsov).

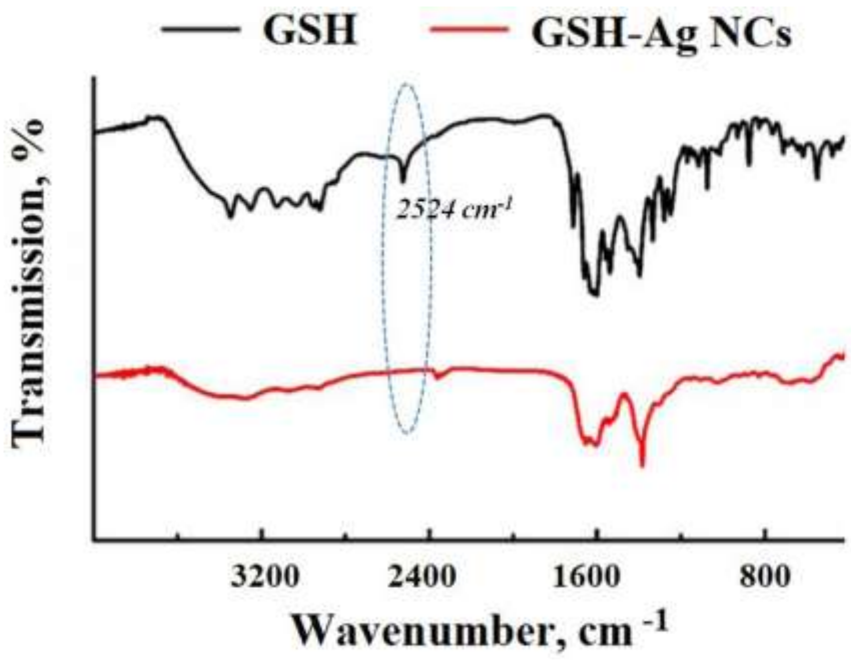

Figure S1. FTIR spectra of GSH and GSH-Ag NCs. The S−H stretching vibration at 2524 cm-1 in free GSH is absent in the GSH-Ag NCs. GSH features in the region 2000-400 cm-1 confirm the presence of GSH protection of the nanocluster.

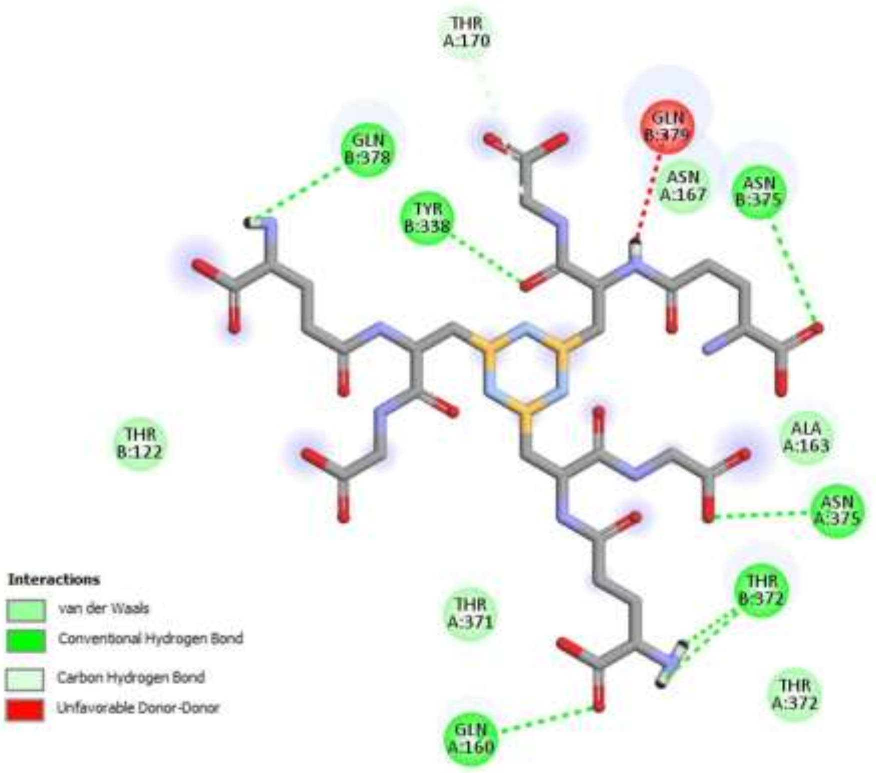

Figure S2. 2D interactions of Ag-SG NCs with amino acid residues of the conserved entrance aperture of TolC.

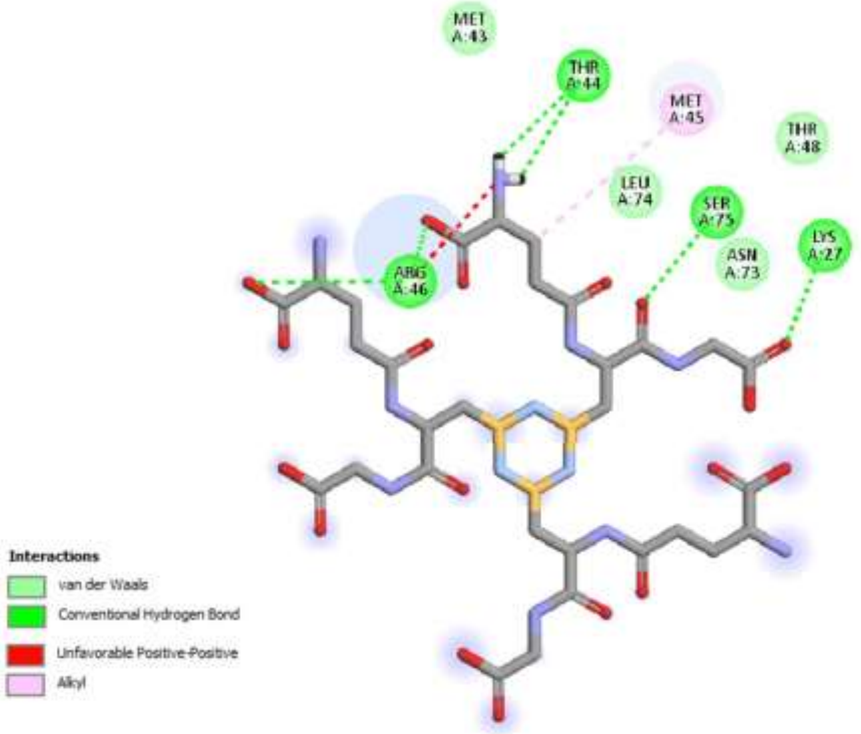

Figure S3. 2D interactions of Ag-SG NCs within the active site of cation efflux system protein CusF.

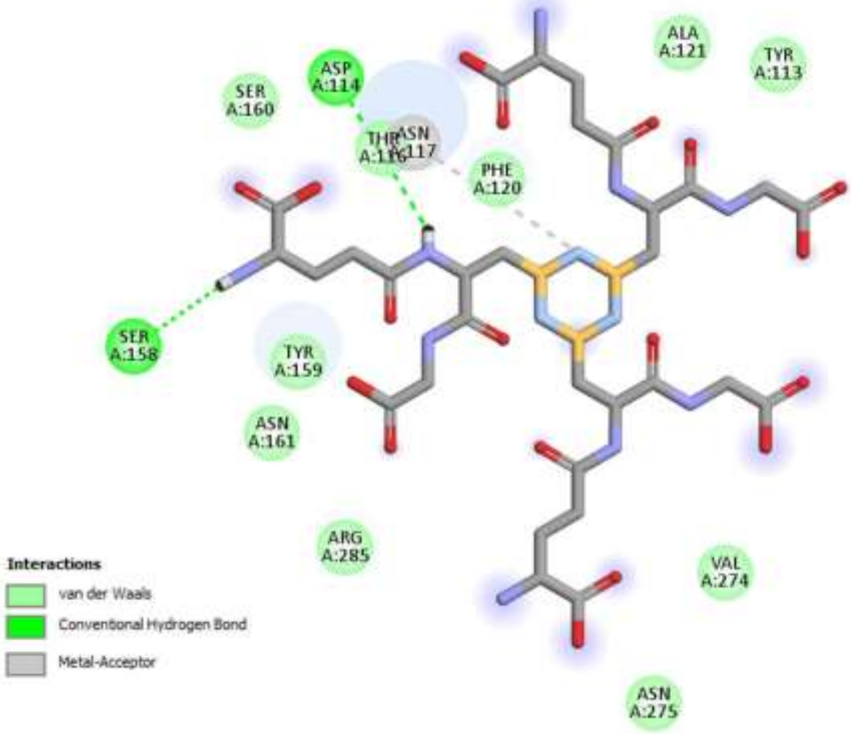

Figure S4. 2D interactions of Ag-SG NCs within the active site of D-alanyl-D-alanine carboxypeptidase.
